# Are single-nucleotide polymorphisms previously linked to inhaled corticosteroid response associated with obese-asthma in children?

**DOI:** 10.1111/pai.14279

**Published:** 2024-11

**Authors:** Cristina Longo, Richard Chiv, Zhongli Xu, Erick Forno, Wei Chen, Andreas Boeck, Raquel Granell, Michael Salvermoser, Bianca Schaub, Juan C. Celedón, Stephen Turner, Susanne Vijverberg, Anke H. Maitland-van der Zee

**Affiliations:** 1 Research Centre, Centre Hospitalier Universitaire Sainte-Justine, Québec, California, USA; 2 Faculty of Pharmacy, University of Montreal, Québec, California, USA; 3 Department of Pulmonary Medicine, Amsterdam UMC, University of Amsterdam, Amsterdam, The Netherlands; 4 Division of Pediatric Pulmonary Medicine, University of Pittsburgh, Pittsburgh, USA; 5 Department of Pediatrics, Indiana University School of Medicine, Indianapolis, Indiana, USA; 6 Pediatric Allergology, Department of Pediatrics, Dr. Von Hauner Children’s Hospital, University Hospital, LMU, Munich, Germany; 7 Member of German Center for Lung Research - DZL, LMU, Munich, Germany; 8 Centre for Applied Excellence in Skin and Allergy Research (CAESAR), Centre for Academic Primary Care, Bristol Medical School, University of Bristol; 9 Member of German Center for Child and Adolescent Health-DZKJ, LMU, Munich, Germany; 10 University of Aberdeen, Aberdeen, Scotland, UK; 11 Department of Pediatric Pulmonology and Allergy, Emma Children’s Hospital Amsterdam UMC, University of Amsterdam, Amsterdam, The Netherlands

## To the Editor,

Childhood obesity is an important risk factor for the development of asthma and, in those who have asthma, may reduce their response to conventional treatment as well as worsen the severity and progression of the disease.^1^ This obese-asthma phenotype has become increasingly prevalent, with nearly 20% and 30% of children with asthma being overweight and obese, respectively.^2^

Asthma control can be achieved with short-acting beta-agonists and inhaled corticosteroids (ICS). However, between 40% and 70% of children with asthma remain inadequately controlled and up to 15% of children with asthma experience exacerbations despite treatment with ICS.^3,4^ Among children with asthma, those who are overweight or obese are less likely to respond to ICS and are at a greater risk of exacerbation.^5^ Mechanisms underlying poor asthma outcomes in children with comorbid obesity are thought to be multi-factorial, including abnormal ventilation, chronic inflammation, epigenetics changes that lead to nonatopic inflammation, and genetics.^6^

Previous genome-wide association (GWAS) studies have suggested several potential shared genetic pathways between obesity and asthma in childhood by identifying single-nucleotide polymorphisms (SNPs) associated with both conditions.^7^ Given the link between obesity and reduced ICS response in children with asthma, we hypothesized that some SNPs previously linked to poor ICS response in children will also be associated with obesity, which may highlight potential underlying mechanisms of the pediatric obese-asthma phenotype. To examine this hypothesis, we assessed the association between candidate SNPs that had been previously suggested to be associated with poor ICS response, identified from the discovery phase and enrichment analyses included in a larger GWAS on poor ICS response in children conducted within the Pharmacogenomics in Childhood Asthma consortium (PiCA),^8^ and obesity among children with asthma aged 2–16 years old who were treated with ICS.

This exploratory multicentre candidate SNP study was conducted within the PiCA consortium and included overlapping data sources with the published meta-GWAS on poor ICS response in children,^8^ with poor response defined as any urgent care visit or use of oral corticosteroids for asthma despite treatment with ICS in the previous 12 months. For this candidate SNP study, we included children with asthma who were treated with ICS or ICS combination therapy in four cross-sectional studies and one cohort study conducted between 1993 and 2012: the Pharmacogenetics of Asthma Medication in Children (PACMAN),^9^ the Pediatric Asthma Gene Environment Study (PAGES),^10^ the Hartford-Puerto Rico (HPR) study,^11^ the Clinical Asthma Research Association Study (CLARA),^12^ and the Avon Longitudinal Study of Parents and Children (ALSPAC).^13^ In all studies, we extracted data on age, sex, weight, height, ethnicity, second-hand smoke exposure, asthma severity, and atopy. All studies received approval from their local institutional review boards and written consent forms were obtained from participating children’s parents.^9–13^

Asthma was either self-reported (PACMAN and ALSPAC) or diagnosed by a physician (PAGES and CLARA). In the HPR study, the physician-confirmed diagnosis of asthma (with at least one episode of wheeze in the previous year) was reported by the children’s parents. ICS use was defined using pharmacy or medical claims (PACMAN and CLARA, respectively) or self-reported questionnaires (PAGES, H-PR, and ALSPAC). Children with cystic fibrosis and/or bronchopulmonary dysplasia, missing data on height and/or weight, who did not provide consent for DNA sampling or with poor quality control were excluded.

Of the list of 110 suggestive SNPs for poor ICS response in children with asthma identified in a previously published meta-GWAS in PiCA (see Table S1 for SNP attributes),^8^ only 29 in HPR/CLARA, 11 in PACMAN/PAGES, and 6 in ALSPAC were genotyped, respectively, while the remaining SNPs were imputed with high quality (*r*^2^ > 90%). Genotyping and/or imputation procedures for each study have been previously described.^8,12,14,15^ From this list of 110 candidate SNPs, we retained only those not considered to be in linkage disequilibrium (defined as an r^2^ > 0.20) with a minor allele frequency (MAF) ≥5%. Among the listed SNPs with MAF≥5% considered to be in linkage disequilibrium, the available SNP having the strongest association with poor ICS response from the meta-GWAS was selected as a candidate SNP for further analysis.

Our primary outcome was obesity status defined by computing the children’s body mass index (BMI) using the height and weight available in parental questionnaires (PACMAN and PAGES) or measured at baseline during clinical assessment periods (HPR, CLARA, and ALSPAC). BMI values were transformed into age- and sex-specific BMI z-scores and associated percentiles based on the World Health Organization (WHO) growth charts for children aged 2–4 and 5–19 years old. Obesity was defined as a BMI greater than the 95th percentile. The outcome was assessed at cohort entry prior to index ages 4, 8, 10, 12, 14, and 16 years for ALSPAC or at enrolment date for cross-sectional studies (PACMAN, CLARA, PAGES, and HPR).

Logistic regression was used for the multivariable analysis of the candidate SNPs and obesity in each study, adjusting for age, sex, and the first 10 principal components to adjust for population stratification derived from genotypic data. The estimated odds ratios (ORs) with their respective 95% confidence intervals (95% CI) for each SNP were then meta-analyzed across studies using a random effects model. The Benjamini–Hochberg procedure was used to adjust for multiple testing, with a false discovery rate (FDR) of 20% considered acceptable. All statistical analysis was performed using R Statistical Software (version 4.3.1, R Foundation for Statistical Computing, Vienna, Austria).

**FIGURE 1:**
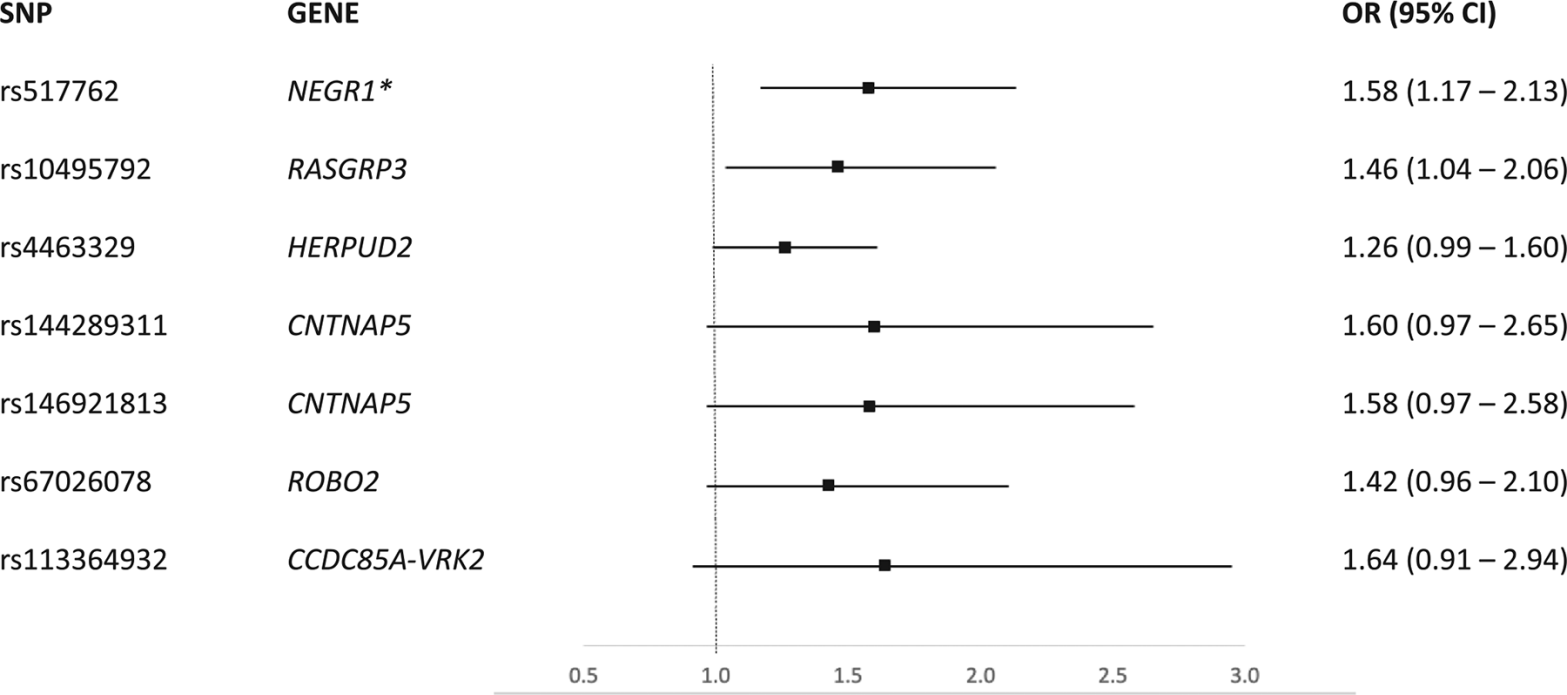
Odds ratio (OR) with 95% confidence intervals for obesity among those with and without SNPs associated with poor response to inhaled corticosteroids from the meta-analysis. *Met statistical significance threshold, defined as a *p*-value (after false discovery rate correction) < 0.2 Of the SNPs suggestively associated with obesity, only rs10495792 in the *RASGRP3* gene was genotyped in CLARA and HPR, but not in ALSPAC, PACMAN, or PAGES, while the rest were imputed.

This study included a total of 1,511 children with asthma treated with ICS. Overall, there were 318 obese children (21%). Included children were on average older at enrolment (9 years old, ±2.7), male (57%), mostly atopic (84%) with mild-to-moderate asthma (91%). There were more children who had moderate and severe asthma in the PAGES (61% and 16%) and CLARA (52% and 42%) compared to other included studies (12% and 2%). Overall, 36% of children with asthma experienced an exacerbation despite being treated with ICS. Of the 56 retained SNPs, 7 were associated with obesity using a nominal p-value (Figure 1) but only the SNP rs517762 in the neuronal growth regulator 1 gene (*NEGR1*) remained significantly associated after adjustment for multiple testing (pooled OR 1.58 [95% CI 1.17–2.13], heterogeneity p-value 0.45).

Our findings in this exploratory study suggest that the SNP rs517762 in the *NEGR1* gene, which has been previously shown to be implicated in poor ICS response, is also associated with obesity in children with asthma treated with ICS. Our results are consistent with and expand on those of a prior work of an association between *NEGR1* variants and both obesity and asthma in a previous GWAS.^7,16^ Moreover, our findings also support those of a previously reported association between leptin (satiety hormone) and obesity, lower respiratory function and asthma exacerbations, given that *NEGR1* regulates leptin function.^17^ One possible mechanism underlying reduced ICS response in obese children with asthma is the secretion of proinflammatory cytokines and mediators (including leptin) in adipose tissues. Leptin can stimulate the activation of monocytes, CD4^+^ and CD8^+^ T cells and induce the production of pro-inflammatory cytokines (e.g., TNF-α and interleukins-18), which have been shown previously to be implicated in the pathophysiology of asthma.^17^

We recognize several study limitations. First, we lacked statistical power to detect SNPs with weak to modest effects on obesity, given our sample size. Due to this lack of power, we were unable to explore whether asthma severity indicators, including ICS dosage, could act as effect modifiers for the candidate SNP–obesity relationships investigated in this study. Second, we were unable to establish temporal relationships as most studies included in this analysis were cross-sectional. Whether obesity was a cause or consequence of poor ICS response in the included studies is unknown. Thus, results should be interpreted as hypothesis-generating only. Third, data for asthma were mostly parent-reported (questionnaires), though asthma definitions have been validated and are commonly used in epidemiologic studies. Fourth, age- and sex-adjusted BMIz and associated percentiles were used to classify obesity status in children with asthma. While this approach was shown to be an adequate screening tool for high adiposity in children aged 9 years old and over,^18^ systematic reviews have shown that it is often highly specific but moderately sensitive, leading to nondifferential misclassification of obesity status, particularly in younger children.^19^ We, therefore, expect this measurement error to lead to less precise estimates and bias toward the null. Fifth, we did not reevaluate the association between the selected candidate SNPs and poor ICS response since our study used data sources that had been previously included in the published GWAS on poor ICS response in children with asthma. Nevertheless, this GWAS showed that the *NEGR1* gene, which was identified through gene set enrichment, had a clinically relevant association with poor ICS response in European children with asthma (OR 1.39; 95%CI 1.18–1.63).^8^ Last, we did not have access to epigenetics in the data sources used for this study. We recognize that epigenetic changes, such as hypomethylation of T-cell signaling and macrophage activation genes, may also drive the obese-asthma phenotype in children,^6,20^ and evaluating this could shed further light regarding potential mechanisms that could be used for targeted therapeutics.

In summary, this multicenter candidate SNP study may suggest a possible role of SNP rs517762 in *NEGR1* in the obese-asthma phenotype in children. Prospective and functional studies should further validate these findings. These studies could also explore models that include different SNPs or genes interactions.

## Supplementary Material

Online supplement

SUPPORTING INFORMATION

Additional supporting information can be found online in the Supporting Information section at the end of this article.
