# Supplementary material for: Are single-nucleotide polymorphisms previously linked to inhaled corticosteroid response associated with obese-asthma in children?
Source: Pediatr Allergy Immunol. Author manuscript; Available in PMC 2026 Jan 20. (PMC12817123; doi:10.1111/pai.14279)
Supplement: Online supplement [file NIHMS2137124-supplement-Online_supplement.docx]

**SUPPLEMENTARY MATERIAL**

**Table S1- Attributes for list of SNPs suggestively associated with poor ICS response in Meta-GWAS**

| RS Number | Nearby Gene | Chromosome | Consequence | Risk Allele | Global Risk Allele Frequency (RAF) | Association with Poor ICS Response | |
| --- | --- | --- | --- | --- | --- | --- | --- |
|  |  |  |  |  |  | Log(OR) | SE |
| rs1814054 | *NEGR1* | 1 | Intron | T | 0.240 | -0.308 | 0.079 |
| rs517762 | *NEGR1* | 1 | Intron | T | 0.793 | 0.329 | 0.082 |
| rs35245473 | *ZNF648-GLUL* | 1 | None | C | 0.102 | 0.468 | 0.100 |
| rs71632139 | *ZNF648-GLUL* | 1 | None | C | 0.082 | 0.468 | 0.100 |
| rs7575244 | *LTBP1* | 2 | *Intron* | T | 0.418 | 0.012 | 0.147 |
| rs11681246 | *LTBP1* | 2 | *Intron* | G | 0.394 | -0.327 | 0.070 |
| rs631004 | *LTBP1* | 2 | *Intron* | C | 0.305 | 0.224 | 0.073 |
| rs10495792 | *RASGRP3* | 2 | *Intron* | A | 0.087 | 0.290 | 0.260 |
| rs4953253 | *PRKCE* | 2 | *Intron* | A | 0.745 | 0.280 | 0.172 |
| rs6738524 | *PRKCE* | 2 | *Intron* | T | 0.747 | 0.257 | 0.175 |
| rs56310656 | *EPAS1* | 2 | *Intron* | A | 0.141 | 0.312 | 0.103 |
| rs6545073 | *FSHR* | 2 | *None* | A | 0.367 | 0.224 | 0.070 |
| rs7569759 | *NRXN1* | 2 | *Intron* | A | 0.140 | -0.329 | 0.075 |
| rs7569775 | *NRXN1* | 2 | *Intron* | C | 0.260 | -0.328 | 0.075 |
| rs113364932 | *CCDC85A-VRK2* | 2 | *None* | A | 0.016 | 0.788 | 0.160 |
| rs72805125 | *CCDC85A-VRK2* | 2 | *None* | T | 0.039 | 0.737 | 0.158 |
| rs76496334 | *CNTNAP5* | 2 | *Intron* | T | 0.015 | 0.827 | 0.169 |
| rs146921813 | *CNTNAP5* | 2 | *Intron* | C | 0.010 | 0.818 | 0.169 |
| rs141194780 | *CNTNAP5* | 2 | *Intron* | A | 0.034 | 0.818 | 0.169 |
| rs144289311 | *CNTNAP5* | 2 | *Intron* | A | 0.031 | 0.844 | 0.170 |
| rs145694710 | *CNTNAP5* | 2 | *Intron* | T | 0.035 | 0.822 | 0.169 |
| rs17011852 | *CNTNAP5* | 2 | *Intron* | G | 0.032 | 0.843 | 0.170 |
| rs3732279 | *UPP2* | 2 | *3 Prime UTR Variant* | C | 0.073 | 0.561 | 0.299 |
| rs10168221 | *MYO3B* | 2 | *Intron* | C | 0.447 | 0.277 | 0.067 |
| rs6756607 | *MYO3B* | 2 | *Intron* | G | 0.540 | -0.281 | 0.067 |
| rs2465662 | *AOX1* | 2 | *Intron* | C | 0.296 | 0.123 | 0.195 |
| rs7587871 | *AOX1* | 2 | *Intron* | A | 0.318 | 0.086 | 0.190 |
| rs7420798 | *AOX1* | 2 | *Intron* | G | 0.347 | 0.085 | 0.189 |
| rs12988162 | *AOX1* | 2 | *Intron* | A | 0.349 | 0.080 | 0.188 |
| rs10208193 | *PLEKHM3* | 2 | *Intron* | A | 0.276 | 0.313 | 0.073 |
| rs13388101 | *PLEKHM3* | 2 | *Intron* | G | 0.263 | 0.309 | 0.073 |
| rs74896344 | *PLEKHM3* | 2 | *Intron* | C | 0.263 | 0.313 | 0.073 |
| rs789 | *RBMS3* | 3 | *Intron* | A | 0.352 | 0.296 | 0.071 |
| rs6549930 | *RBMS3* | 3 | *Intron* | C | 0.446 | 0.295 | 0.068 |
| rs67026078 | *CACNA2D3-WNT5A* | 3 | *Intron* | C | 0.061 | 0.408 | 0.246 |
| rs35635902 | *CACNA2D3-WNT5A* | 3 | *Intron* | C | 0.039 | 0.384 | 0.240 |
| rs1166980 | *ROBO2* | 3 | *Intron* | G | 0.184 | -0.028 | 0.088 |
| rs6772804 | *ROBO2* | 3 | *Intron* | T | 0.278 | 0.022 | 0.157 |
| rs9864605 | *ROBO2* | 3 | *Intron* | T | 0.277 | 0.015 | 0.153 |
| rs7618319 | *ROBO2* | 3 | *Intron* | T | 0.330 | 0.021 | 0.151 |
| rs72891542 | *ROBO2* | 3 | *Intron* | T | 0.057 | 1.439 | 0.350 |
| rs72891545 | *ROBO2* | 3 | *Intron* | A | 0.057 | 1.567 | 0.361 |
| rs80109563 | *ROBO2* | 3 | *Intron* | T | 0.031 | 1.852 | 0.458 |
| rs77698848 | *ROBO2* | 3 | *Intron* | A | 0.052 | 1.802 | 0.480 |
| rs75844835 | *ROBO2* | 3 | *Intron* | G | 0.012 | 2.199 | 0.556 |
| rs75804244 | *ROBO2* | 3 | *Intron* | A | 0.042 | 1.867 | 0.439 |
| rs79222358 | *ROBO2* | 3 | *Intron* | G | 0.032 | 1.477 | 0.411 |
| rs74702233 | *ROBO2* | 3 | *Intron* | T | 0.001 | 1.425 | 0.406 |
| rs79752754 | *ROBO2* | 3 | *Intron* | C | 0.011 | 1.425 | 0.406 |
| rs12629167 | *ROBO2* | 3 | *Intron* | C | 0.037 | 1.425 | 0.406 |
| rs7612655 | *ROBO2* | 3 | *Intron* | T | 0.030 | 1.425 | 0.406 |
| rs7613086 | *ROBO2* | 3 | *Intron* | T | 0.046 | 1.425 | 0.406 |
| rs75336627 | *ROBO2* | 3 | *Intron* | A | 0.043 | 1.600 | 0.420 |
| rs77225325 | *ROBO2* | 3 | *Intron* | A | 0.032 | 1.600 | 0.420 |
| rs76099377 | *ROBO2* | 3 | *Intron* | G | 0.043 | 1.600 | 0.420 |
| rs7623806 | *ROBO2* | 3 | *Intron* | C | 0.043 | 1.600 | 0.420 |
| rs72891555 | *ROBO2* | 3 | *Intron* | G | 0.032 | 1.598 | 0.440 |
| rs838642 | *SLC9A9* | 3 | *Intron* | G | 0.445 | 0.299 | 0.069 |
| rs67979603 | *ARHGAP24* | 4 | *None* | A | 0.502 | -0.076 | 0.133 |
| rs62315647 | *ARHGAP24* | 4 | *Intron* | C | 0.026 | 0.803 | 0.236 |
| rs268529 | *SEMA5A* | 5 | *Intron* | C | 0.327 | 0.289 | 0.074 |
| rs707637 | *SEMA5A* | 5 | *Intron* | T | 0.176 | 0.364 | 0.090 |
| rs10473694 | *CDH10* | 5 | *Intron* | A | 0.384 | 0.297 | 0.072 |
| rs10473695 | *CDH10* | 5 | *Intron* | A | 0.384 | 0.297 | 0.072 |
| rs17459974 | *CDH10* | 5 | *Intron* | C | 0.238 | 0.299 | 0.072 |
| rs434309 | *ZNF608-GRAMD3* | 5 | *Intron* | A | 0.356 | 0.298 | 0.111 |
| rs444610 | *ZNF608-GRAMD3* | 5 | *Intron* | A | 0.429 | 0.304 | 0.112 |
| rs13203042 | *LAMA2* | 6 | *Intron* | G | 0.390 | -0.282 | 0.069 |
| rs12527452 | *LAMA2* | 6 | *Intron* | A | 0.388 | -0.314 | 0.106 |
| rs9397300 | *NOX3-ARID1B* | 6 | *Intron* | A | 0.353 | 0.301 | 0.071 |
| rs2493700 | *NOX3-ARID1B* | 6 | *Intron* | G | 0.485 | -0.336 | 0.069 |
| rs6921718 | *PDE10A* | 6 | *Intron* | C | 0.176 | 0.356 | 0.089 |
| rs57042153 | *PDE10A* | 6 | *Intron* | T | 0.173 | 0.358 | 0.089 |
| rs11767474 | *TBX20* | 7 | *None* | C | 0.190 | -0.336 | 0.086 |
| rs4463329 | *HERPUD2* | 7 | *Intron* | G | 0.582 | -0.256 | 0.065 |
| rs62454596 | *HERPUD2* | 7 | *Intron* | C | 0.396 | 0.256 | 0.065 |
| rs138763940 | *RUNX1T1* | 8 | *None* | T | 0.004 | 0.067 | 0.511 |
| rs1977324 | *CXCL12* | 10 | *Intron* | C | 0.699 | 0.287 | 0.120 |
| rs12780983 | *JMJD1C* | 10 | *Intron* | T | 0.231 | 0.282 | 0.072 |
| rs35468796 | *JMJD1C* | 10 | *Intron* | G | 0.143 | 0.282 | 0.072 |
| rs487926 | *KCNMA1* | 10 | *Intron* | A | 0.237 | 0.116 | 0.178 |
| rs571396 | *KCNMA1* | 10 | *Intron* | G | 0.326 | 0.145 | 0.169 |
| rs56368530 | *TEAD1* | 11 | *Intron* | G | 0.126 | 0.408 | 0.101 |
| rs61746574 | *TPCN2* | 11 | *Missense* | A | 0.122 | 0.004 | 0.246 |
| rs79481933 | *TPCN2* | 11 | *Intron* | A | 0.029 | 0.093 | 0.238 |
| rs1453830 | *OPCML* | 11 | *Intron* | A | 0.544 | 0.251 | 0.069 |
| rs514075 | *OPCML* | 11 | *Intron* | G | 0.881 | 0.486 | 0.114 |
| rs78501135 | *TMTC1* | 12 | *Intron* | C | 0.099 | 0.447 | 0.106 |
| rs7954923 | *TMTC1* | 12 | *Intron* | A | 0.885 | -0.441 | 0.107 |
| rs224589 | *SLC11A2* | 12 | *Intron* | G | 0.752 | 0.318 | 0.077 |
| rs440595 | *SLC11A2* | 12 | *Intron* | A | 0.747 | 0.320 | 0.077 |
| rs12825767 | *CPM* | 12 | *Intron* | G | 0.049 | -0.611 | 0.166 |
| rs1695154 | *CPM* | 12 | *Intron* | G | 0.565 | -0.288 | 0.070 |
| rs10145080 | *RTN1* | 14 | *Intron* | T | 0.240 | 0.318 | 0.072 |
| rs1952032 | *RTN1* | 14 | *Intron* | C | 0.420 | 0.318 | 0.072 |
| rs2291355 | *ATP10A* | 15 | *Intron* | A | 0.399 | -0.279 | 0.071 |
| rs7177340 | *ARNT2* | 15 | *Intron* | C | 0.183 | 0.299 | 0.075 |
| rs11856226 | *ARNT2* | 15 | *Intron* | T | 0.295 | 0.305 | 0.075 |
| rs4996904 | *RGMA* | 15 | *Intron* | A | 0.251 | -0.294 | 0.074 |
| rs12440283 | *MCTP2* | 15 | *Intron* | G | 0.600 | -0.225 | 0.069 |
| rs11858617 | *SPATA8-ARRDC4* | 15 | *None* | T | 0.057 | 0.627 | 0.144 |
| rs72759231 | *SPATA8-ARRDC4* | 15 | *None* | G | 0.062 | 0.677 | 0.140 |
| rs28761328 | *DLGAP1-ZBTB14* | 18 | *None* | A | 0.147 | 0.445 | 0.097 |
| rs9951179 | *DLGAP1-ZBTB14* | 18 | *None* | A | 0.136 | 0.439 | 0.097 |
| rs10164193 | *ASXL3* | 18 | *Intron* | G | 0.101 | 0.501 | 0.123 |
| rs7227658 | *ASXL3* | 18 | *Intron* | A | 0.049 | 0.475 | 0.121 |
| rs233900 | *ADAMTS5* | 21 | *Intron* | T | 0.262 | 0.246 | 0.116 |
| rs233899 | *ADAMTS5* | 21 | *Intron* | A | 0.262 | 0.246 | 0.116 |
| rs695783 | *SLC25A18* | 22 | *Intron* | A | 0.684 | -0.277 | 0.130 |
| rs425676 | *SLC25A18* | 22 | *Intron* | T | 0.487 | -0.277 | 0.133 |
